# Synergistic Sn-Induced Band Convergence in Mn-Doped p-Type PbTe Enables High Thermoelectric Performance

**DOI:** 10.3390/ma19101947

**Published:** 2026-05-09

**Authors:** Zhilong Zhao, Xiang An, Fan Feng, Jiaxing Luo, Zijian Lin, Chuke Zhao, Ran Ang

**Affiliations:** 1 Key Laboratory of Radiation Physics and Technology, Ministry of Education, Institute of Nuclear Science and Technology, Sichuan University, Chengdu 610064, China; zhao@stu.scu.edu.cn (Z.Z.); axwinner@163.com (X.A.); ff001025@163.com (F.F.); gnixaijl@163.com (J.L.); 13970966083@163.com (Z.L.); zchuke2000@163.com (C.Z.); 2 College of Physics, Sichuan University, Chengdu 610064, China; 3 Institute of New Energy and Low-Carbon Technology, Sichuan University, Chengdu 610065, China

**Keywords:** thermoelectric, PbTe, band convergence, Mn/Sn co-doping, lattice thermal conductivity, figure of merit

## Abstract

**Highlights:**

- Co-doping of Mn and Sn in p-type PbTe is for the first time demonstrated to synergistically promote the dissolution of Na into the matrix.
- An exceptional peak *zT* of ~2.2 at 823 K is achieved, alongside the highest reported room-temperature region *zT* of ~0.4 in this material system.
- The co-doping strategy significantly enhances the convergence of *L* and *Σ* valence bands, leading to a substantial increase in the density-of-states effective mass.
- Multiscale lattice defects induced by co-doping suppress lattice thermal conductivity to near the theoretical minimum (~0.5 W m^−1^ K^−1^).
- The synergistic Mn and Sn co-doping strategy provides an effective pathway to decouple interdependent electrical and thermal transport parameters.
- Achieving both a high peak and a high average *zT* advances PbTe-based materials for practical mid-temperature waste-heat recovery.
- The enhanced Na solubility via Sn-induced Pb vacancies offers a new perspective for optimizing doping efficiency in PbTe systems.

**Abstract:**

The inherent coupling of electrical and thermal transport parameters poses a significant challenge for enhancing the thermoelectric figure of merit (*zT*) in PbTe-based materials. Herein, we report a synergistic co-doping strategy employing Mn and Sn in p-type PbTe to simultaneously optimize the band structure and suppress lattice thermal conductivity. Sn incorporation not only induces additional Pb vacancies, thereby increasing hole carrier concentration, but also facilitates the enhanced solubility of Na dopants within the matrix, as confirmed by microscopic and compositional analyses. More importantly, the cooperative effect of Mn and Sn substantially enhances convergence between the *L* and *Σ* valence bands, leading to an increased density-of-states effective mass and a pronounced enhancement of the Seebeck coefficient. Meanwhile, multiscale lattice defects introduced by co-doping effectively scatter phonons over a broad frequency spectrum, reducing the lattice thermal conductivity to near the theoretical minimum (~0.5 W m^−1^ K^−1^). As a result, the Pb_0.91−*x*_Na_0.04_Mn_0.04_Sn*_x_*Te system achieves an exceptional peak *zT* of ~2.2 at 823 K, a high room-temperature *zT* of ~0.4, and a favorable average *zT* of ~1.3 over the temperature range of 303–823 K. Notably, the room-temperature *zT* of ~0.4 represents the highest value reported to date for p-type PbTe in the room-temperature region. This work demonstrates that Mn and Sn co-doping provides a compelling pathway for realizing both high peak and average thermoelectric performance, advancing PbTe-based materials toward practical waste-heat recovery applications.

## 1. Introduction

Thermoelectric materials enable direct conversion of waste heat into electricity, offering a viable approach to alleviate energy scarcity through efficient thermal energy recovery [1,2,3]. The conversion efficiency of a thermoelectric device is determined by the dimensionless figure of merit, defined as *zT* = *S*^2^*σT*/(*κ*_ele_ + *κ*_lat_), where *S*, *σ*, *T*, *κ*_ele_, and *κ*_lat_ denote the Seebeck coefficient, electrical conductivity, absolute temperature, electronic thermal conductivity, and lattice thermal conductivity, respectively [4,5,6]. A key obstacle in advancing *zT* lies in the intricate coupling among these transport parameters, where optimizing one often compromises others [7,8,9]. In contrast, reducing the relatively independent lattice thermal conductivity κlat has proven effective for enhancing *zT* across various material systems [4,10,11].

Among mid-temperature thermoelectric materials (600–900 K), p-type PbTe has garnered substantial interest owing to its favorable electrical transport properties, stemming from the small energy separation (Δ*E_L_*_-*Σ*_) between its *L* and *Σ* valence bands [12,13,14,15,16,17], as well as its intrinsically low *κ*_lat_ originating from heavy Pb atoms and strong lattice anharmonicity [18]. It is well recognized that Na doping in PbTe not only optimizes carrier concentration but also promotes convergence of the *L* and *Σ* bands, leading to an enhanced Seebeck coefficient and a peak *zT* of approximately 1.8 [19]. However, the limited solubility of Na in PbTe constrains its doping efficiency and further band engineering [20]. In our recent work, we demonstrated that introducing Pb vacancies effectively increases Na solubility [20], and subsequent Mn doping further promotes Na dissolution [21,22], optimizes valence band convergence, and introduces multiscale defects that collectively suppress *κ*_lat_ [23]. These efforts resulted in a high peak *zT* of ~1.9 in the Pb_0.91_Na_0.04_Mn_0.04_Te system, highlighting the effectiveness of this strategy [24,25].

**Figure 1:**
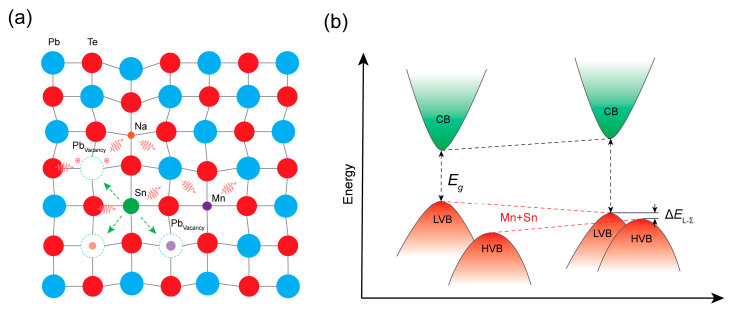
(**a**) Schematic illustration of lattice distortion and enhanced solubility of Mn and Na induced by Na, Mn, and Sn co-doping (The arrow on the waveform represents a phonon). (**b**) Schematic illustration of band convergence induced by Mn and Sn co-doping in p-type PbTe.

Building upon these findings, we now introduce Sn as an additional dopant into the Pb_0.91_Na_0.04_Mn_0.04_Te matrix to further enhance the thermoelectric performance. As schematically depicted in Figure 1a, the incorporation of Sn, Mn, and Na induces local lattice distortions that intensify phonon scattering [25]. Notably, Sn substitution at Pb sites promotes the formation of additional Pb vacancies, each contributing two hole carriers, thereby increasing the hole concentration and further facilitating Na dissolution into the matrix—a finding corroborated by scanning electron microscopy (SEM) and energy-dispersive X-ray spectroscopy (EDS) analyses. Moreover, Figure 1b illustrates the synergistic effect of Mn and Sn co-doping on the valence band structure, where the convergence between the *L* and *Σ* bands is further strengthened compared with single-element Sn or Mn doping [25]. This enhanced band convergence significantly elevates the density-of-states effective mass and, consequently, the Seebeck coefficient. Benefiting from these synergistic effects, the Pb_0.91−*x*_Na_0.04_Mn_0.04_Sn*_x_*Te series achieves a room-temperature *zT*_303K_ of ~0.4, a peak *zT* of ~2.2 at 823 K, and a favorable average *zT*_avg_ of ~1.3 across the entire temperature range. The lattice thermal conductivity was maintained below 0.5 W m^−1^ K^−1^, approaching the theoretical minimum. These results demonstrate that Mn and Sn co-doping provides a compelling pathway for realizing both high peak and average thermoelectric performance in PbTe-based materials, advancing their potential for practical waste-heat recovery applications.

## 2. Materials and Methods

### 2.1. Material Synthesis

Polycrystalline Pb_0.91−*x*_Na_0.04_Mn_0.04_Sn*_x_*Te (*x* = 0, 0.005, 0.01, 0.02, 0.03, 0.04) was synthesized by melting stoichiometric elements of lead (Pb: 99.999%), tellurium (Te: 99.9999%), sodium (Na: 99.95%), manganese (Mn, 99.999%) and tin (Sn: 99.999%) (Manufactured by Zhongnuo Advanced Materials Technology Co., Ltd., Beijing, China) in an evacuated fused silica tube (∼10^−4^ Torr) at 1273 K for 6 h and was then quenched in cold water. The obtained ingots were annealed at 923 K for 3 days and quenched in cold water. Then the obtained ingots were ground in a glove box with a mortar and then pressed into uniform dense sheets by hot pressing (HP, Home-made, Tongji University, Shanghai, China). The HP mechanism was installed with an induction heating system at 873 K for 15 min under a 55 MPa uniaxial pressure. The resulting disc-shaped samples were 12.7 mm in diameter, which was determined by the Archimedes method.

### 2.2. Phase and Composition Characterization

The X-ray diffraction (XRD) measurements were performed with Cu K_α_ radiation at room temperature. The morphological, structural and chemical characteristics of the synthesized samples were investigated by scanning electron microscopy (SEM; JEOL 7100, Nippon Electronics Co., Ltd., Nagoya, Japan equipped with electron backscatter diffraction detector).

### 2.3. Thermoelectric Property Measurement

The HP manufactured samples were polished into multiple sizes to measure thermoelectric transport properties. The temperature-dependent electrical conductivities *σ* and *S* were measured using a CTA Pro instrument (Beijing Cryoall Science and Technology Co., Ltd., Beijing, China). The Hall mobility *μ*_H_ and carrier concentration *n*_H_ were measured by the Van der Pauw technique under a reversible magnetic field of 1.5 T in high-purity N_2_. The thermal diffusivity (*D*) of a wafer with an average diameter of ~12.7 mm with a thickness of ~1.5 mm relative to temperature was measured by laser flash method (Netzsch, LFA 467 Pro, NETZSCH-Gerätebau GmbH, Selb, Germany). Additionally, thermal conductivity κ_tot_ was computed using the formula *κ*_tot_ = *dDC*_P_, where d is the density measured (shown in Table S1) and *C*_P_ is the heat capacity, which is determined by *C*_P_ (*k*_B_/atom) = [3.07 + 0.00047(*T*/*K* − 300)] for lead chalcogenides and is obtained by fitting the experimental data reported by Blachnik within an uncertainty of ±2% for all the lead chalcogenides at *T* > 300 K. Considering the uncertainty of ±10% in thermal conductivity, the uncertainties for the Seebeck coefficient and electrical resistivity are typically ±5% and ±3%.

## 3. Results

### 3.1. Phase and Microstructure

**Figure 2:**
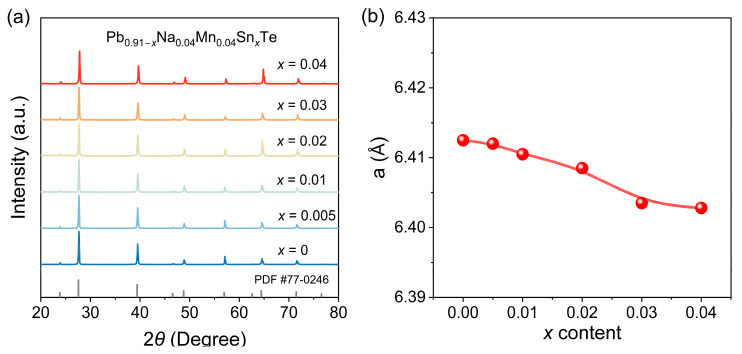
(**a**) Powder X-ray diffraction pattern of Pb_0.91−*x*_Na_0.04_Mn_0.04_Sn*_x_*Te (*x* = 0, 0.005, 0.01, 0.02, 0.03, 0.04). (**b**) Lattice parameters obtained by Rietveld full-spectrum pseudo-orthogonal method.

We performed X-ray diffraction (XRD) measurements on the powdered samples of the synthesized Pb_0.91−*x*_Na_0.04_Mn_0.04_Sn*_x_*Te (*x* = 0, 0.005, 0.01, 0.02, 0.03, 0.04) series at room temperature. Figure 2a presents the phase and crystal structure information of these samples. The main diffraction peaks of all Sn-containing samples were in good agreement with the standard PDF#77-0246 card, confirming that the synthesized samples adopted the NaCl-type rock-salt structure and that the PbTe-based materials were successfully prepared. Within the detection range, no secondary phases were observed. To investigate the evolution of the lattice upon Sn incorporation, the lattice parameters were obtained via Rietveld refinement. As shown in Figure 2b, the lattice parameter decreases with increasing Sn concentration. This reduction is attributed to the substitution of Sn at the Pb site, as the ionic radius of Sn^2+^ (0.93 Å) is smaller than that of Pb^2+^ (1.19 Å). The possibility of Sn substituting at the Mn site is ruled out, because the lattice parameter of Sn-containing samples is even smaller than that of Mn-containing samples (~0.8 Å for Mn substitution); if Sn were to replace Mn, the lattice constant would be expected to increase. Moreover, the solubility limit of Mn in PbTe is approximately 12% [26,27,28], which is well above the doping concentration used in this study.

**Figure 3:**
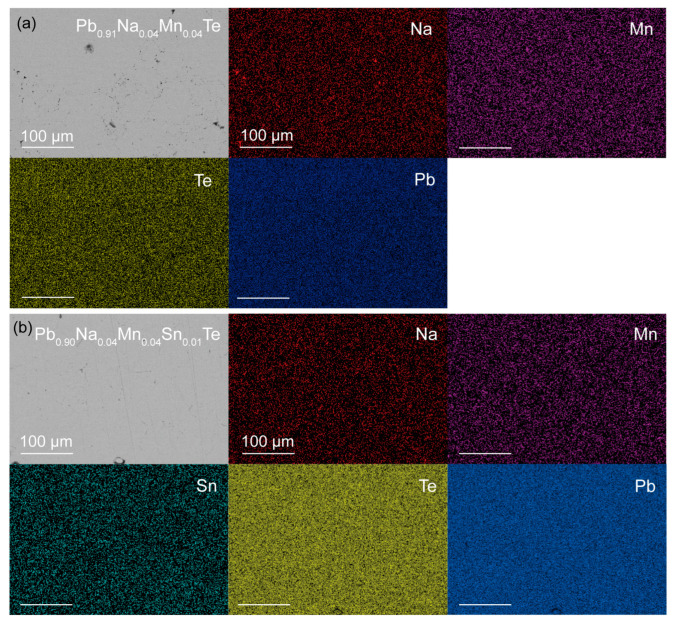
SEM image and corresponding EDS mapping image for (**a**) Pb_0.91_Na_0.04_Mn_0.04_Te and (**b**) Pb_0.90_Na_0.04_Mn_0.04_Sn_0.01_Te samples.

To further investigate the microstructural evolution, scanning electron microscopy (SEM) characterization was performed on two representative samples: Pb_0.91_Na_0.04_Mn_0.04_Te and Pb_0.90_Na_0.04_Mn_0.04_Sn_0.01_Te, as shown in Figure 3a,b. As shown in Figure 3a, at a scale of 100 μm, the polished surface of the sample exhibits a small number of suspected precipitate phases. Corresponding energy-dispersive X-ray spectroscopy (EDS) mapping reveals that the four elements—Na, Mn, Pb, and Te—are predominantly homogeneously distributed, with only a minor presence of Na- and Mn-rich precipitates. Figure 3b presents the sample after Sn doping. Under the same scale, the surface appears notably more uniform, with virtually no observable precipitates. The corresponding EDS elemental maps confirm the homogeneous distribution of all five elements, including Sn. Notably, the originally enriched Na is fully dissolved into the matrix, yielding a homogeneous doping state that not only improves sample uniformity but also strengthens long-term material stability, which is essential for reliable thermoelectric performance. This phenomenon is attributed to the fact that Sn significantly reduces the formation energy of Pb vacancies in the PbTe system, leading to the generation of additional Pb vacancies. Consequently, some of the enriched Na atoms are incorporated into these Pb vacancies, thereby increasing the solubility of Na in the matrix. 

### 3.2. Thermoelectric Properties

**Figure 4:**
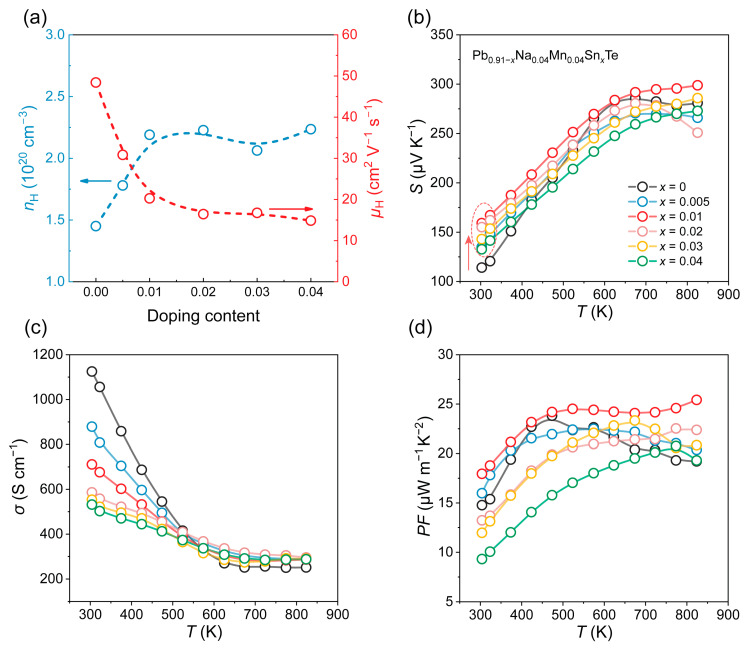
(**a**) Hall carrier concentration (*n*_H_) and Hall carrier mobility (*μ*_H_) of Pb_0.91−*x*_Na_0.04_Mn_0.04_Sn*_x_*Te (*x* = 0, 0.005, 0.01, 0.02, 0.03, 0.04) at 300 K. (**b**) Temperature-dependent Seebeck coefficient (*S*), a significant improvement has been highlighted using dashed circle and arrow, (**c**) electrical conductivity (*σ*), and (**d**) power factor (*S*^2^*σ*) of Pb_0.91−*x*_Na_0.04_Mn_0.04_Sn*_x_*Te (*x* = 0, 0.005, 0.01, 0.02, 0.03, 0.04) sample.

The incorporation of Sn plays a critical role in modulating the electrical transport properties of the Pb_0.91_Na_0.04_Mn_0.04_Te matrix. Figure 4a presents the Hall carrier concentration and mobility of the Pb_0.91−*x*_Na_0.04_Mn_0.04_Sn*_x_*Te (*x* = 0, 0.005, 0.01, 0.02, 0.03, 0.04) samples. A substantial increase in carrier concentration is observed upon doping with *x* = 0.01 Sn, reaching a value of approximately 2.2 × 10^20^ cm^−3^. Further increasing the Sn content (*x* > 0.01) results in a nearly constant carrier concentration. Typically, Sn is divalent in PbTe and substitutes for Pb without introducing additional holes. The observed increase in carrier concentration, considering the monovalent and divalent nature of Na and Mn dopants, respectively, along with the microstructural evidence from SEM, can be rationalized as detailed below. While direct evidence from techniques such as positron annihilation spectroscopy or dedicated DFT calculations remains lacking, the mechanism outlined below represents the most plausible hypothesis consistent with the available experimental and theoretical data. Sn incorporation is known to shift the valence band maximum (VBM) to a higher energy, which, according to first-principles studies [29,30], reduces the formation energy of cation vacancies in PbTe. This Sn-induced lowering of the Pb vacancy formation energy promotes an increased density of V_Pb_ in the matrix. Since each Pb vacancy contributes two hole carriers, the net result is an enhanced carrier concentration, consistent with the experimental trends observed in the Sn-containing samples [31,32]. The corresponding physical mechanism is given by the following equation: $\mathrm{Sn}\overset{\mathrm{PbTe}}{\to }{\mathrm{Sn}}_{\mathrm{Pb}}^{\times }+V_{\mathrm{Pb}}^{\prime \prime }+2h^{\cdot }$. Meanwhile, some of the Na atoms that have redissolved into the matrix can also provide a small number of hole carriers upon occupying Pb sites. In the present system, the carrier concentration remains nearly unchanged when the Sn content exceeds *x* = 0.01. This phenomenon may be attributed to the saturation of Pb vacancies as one of the most probable mechanisms once the Sn content exceeds *x* = 0.01; further Sn incorporation is unlikely to induce additional Pb vacancies, although other possibilities such as neutral defect formation cannot be fully ruled out. Under this condition, the lattice site occupation of Na, Mn, and Sn reaches a relatively stable state, and Na atoms are no longer able to supply hole carriers via substitution at Pb sites. Consequently, additional Sn introduced into the matrix tends to simply substitute for Pb without altering the carrier concentration.

The carrier mobility decreases with increasing Sn content at doping concentrations below *x* = 0.01, reaching approximately 15 cm^2^ V^−1^ s^−1^. This decline is primarily attributed to the enhancement of the carrier effective mass induced by Sn doping, which will be further discussed later. Further increasing the Sn concentration does not lead to a continued decrease in mobility, suggesting that the reduction associated with the increased effective mass has essentially reached saturation. Figure S1 presents the carrier mobility alongside the predicted values. The observed deviation—wherein the measured mobility falls consistently below the predicted values—indicates the presence of additional carrier scattering mechanisms not accounted for in the simplified model. These include, but are not limited to, point defect scattering and local strain fluctuations arising from multi-element doping, as well as grain boundary scattering and enhanced ionized impurity scattering associated with the elevated carrier concentration in the heavily doped material. Figure 4b shows the temperature-dependent Seebeck coefficient of the samples with different Sn contents. Compared with the Sn-free matrix, the Seebeck coefficient of Sn-containing samples is significantly enhanced across the entire temperature range considering the enhancement in carrier concentration. At room temperature, the Seebeck coefficient increases from 114 μV K^−1^ for the matrix to 158 μV K^−1^ for the *x* = 0.01 sample, corresponding to an improvement of approximately 40%. Furthermore, at high temperatures, samples with higher Sn content do not outperform the *x* = 0.01 sample. Considering the structural and electronic similarities between SnTe and PbTe, we infer that excessive Sn content may partially suppress the contribution from the *Σ* band. Nevertheless, the beneficial effect of trace Sn on band convergence in the Pb–Na–Te system at low temperatures is evident.

The temperature-dependent electrical resistivity (*ρ*) of the samples is presented in Figure S2 in the Supplementary Materials. At room temperature, *ρ* increases with increasing Sn content. Notably, above 573 K, the resistivity of Sn-containing samples becomes lower than that of the Sn-free matrix. To provide a clearer illustration of the electrical conductivity, Figure 4c displays the *σ* values of all samples. The decrease in *σ* with increasing temperature is characteristic of degenerate semiconductor behavior. At room temperature, *σ* decreases with increasing Sn content, which can be attributed to the reduced carrier mobility. With increasing temperature, the further decrease in carrier mobility no longer dominates the temperature dependence of electrical conductivity. Instead, the enhancement in electrical conductivity arising from the increased carrier concentration becomes more pronounced. This is clearly illustrated by the higher electrical conductivity observed in the Sn-doped samples compared to the Sn-free matrix at temperatures above 573 K. The *σ* values of the four Sn-containing samples are approximately 280 S cm^−1^ at elevated temperatures, providing a favorable foundation for the high-temperature thermoelectric performance discussed later. Figure 4d presents the power factor (*PF* = *S*^2^*σ*). Benefiting from the significantly enhanced Seebeck coefficient, the *x* = 0.01 sample exhibits a higher *PF* than the matrix at room temperature, reaching approximately 18 μW cm^−1^ K^−2^, despite a slight decrease in electrical conductivity. Although the *PF* values of the *x* = 0.02–0.04 samples are slightly lower than those of the matrix at room temperature, they surpass the matrix values in the mid-to-high-temperature range, owing to the favorable *σ* and Seebeck coefficient in this region. This behavior provides a strong basis for the excellent overall thermoelectric performance of this series of samples. 

**Figure 5:**
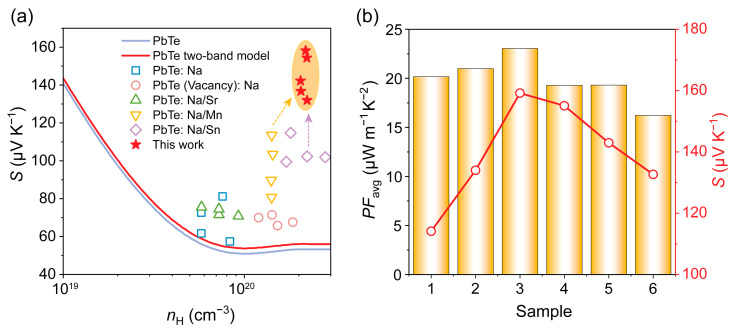
(**a**) Pisarenko analysis for synthesis sample Pb_0.91−**x**_Na_0.04_Mn_0.04_Sn*_x_*Te (*x* = 0, 0.005, 0.01, 0.02, 0.03, 0.04); a significant enhancement in carrier effective mass compared with data in the literature can be concluded. (**b**) Average power factor (*PF*_avg_) and Seebeck coefficients of sample at 303 K.

The Pisarenko plot in Figure 5a clearly demonstrates that Sn doping leads to a higher carrier effective mass, thereby elucidating the origin of the enhanced Seebeck coefficient. The gray line represents the theoretical Pisarenko curve for pristine PbTe, while the purple line corresponds to the theoretical curve based on a two-band model for PbTe. It is evident that the samples in this study (marked region) exhibit higher Seebeck coefficients compared to other reported samples in the Pb–Na–Te system incorporating Sr, Mn, or Sn [7,13,14,23,24]. To elucidate the origin of this enhancement, the density-of-states effective mass (m*) was evaluated for all samples via Hall data under 300 K. With increasing Sn content, the room-temperature m* increases from 1.6 m_e_ to 3 m_e_, representing an approximately 100% enhancement. This substantial increase, taken together with prior reports and our experimental findings, confirms that Sn doping further strengthens the band convergence beyond the level achieved with Mn doping alone. At low temperatures, the Seebeck coefficient is substantially improved by the contribution from the degenerate *L* band [33,34]. Figure 5b presents the compositional dependence of the average power factor (*PF*_avg_) and the average Seebeck coefficient. A strong correlation between the two is observed, confirming that the enhancement in the Seebeck coefficient plays a dominant role in increasing *PF*_avg_. This consistency further substantiates the effectiveness of band convergence induced by Sn doping.

**Figure 6:**
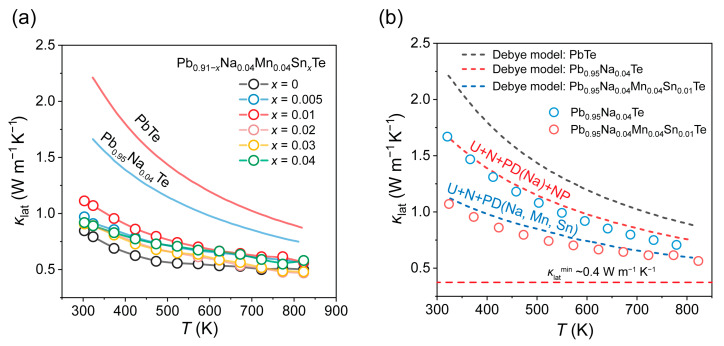
(**a**) Lattice thermal conductivity *κ*_lat_ and (**b**) modified Debye–Callaway model fit for Pb_0.95_Na_0.04_Te and Pb_0.95_Na_0.04_Mn_0.04_Sn_0.01_Te.

The thermal transport properties are closely correlated with the microstructure of the materials. The total thermal conductivity was obtained using the laser flash method to measure the thermal diffusivity and calculated via the formula *κ*_tot_ = *DρC*_P_, where *D* represents the measured thermal diffusivity and *C*_P_ denotes the specific heat capacity. The corresponding data are provided in Figures S3 and S4 in the Supplementary Materials. The electronic thermal conductivity was derived using the Wiedemann–Franz relation [35], *κ*_ele_ = *LσT*, where *L* is the Lorenz number. The lattice thermal conductivity is relatively independent, and generally, lower lattice thermal conductivity is beneficial for enhancing thermoelectric performance. Figure 6a presents the lattice thermal conductivity of the samples. Compared with the Sn-free matrix, the Sn-doped samples exhibit a slight increase in lattice thermal conductivity. However, benefiting from enhanced phonon scattering by the increased Sn-related point defects and the associated lattice strain—which compensates for the increase in lattice thermal conductivity that would otherwise result from the dissolution of secondary phases—the overall lattice thermal conductivity remains relatively low, reaching approximately 0.5 W m^−1^ K^−1^ at high temperatures, which approaches the theoretical minimum of PbTe [36,37,38]. The slight increase in lattice thermal conductivity is primarily attributed to the dissolution of the originally enriched Na- and Mn-containing phases induced by Sn incorporation, which reduces phonon scattering from these precipitates. This observation is consistent with our SEM results. Additionally, the introduction of additional point defects enhances the scattering of mid- and high-frequency phonons, thereby preventing an excessive increase in lattice thermal conductivity and maintaining it at a relatively low level [39,40]. In stark contrast to pristine PbTe and Na-doped PbTe samples, our materials exhibit exceptionally low lattice thermal conductivity, which is favorable for achieving superior thermoelectric performance. To quantitatively assess the contribution of multiscale defects and microstructural features to phonon scattering, a modified Debye model was employed to analyze the phonon transport behavior based on the microstructural observations from SEM [41]. The relevant parameters used in the fitting are summarized in Table S2 in the Supplementary Materials. The relaxation time *τ*_total_ for different scattering mechanisms was combined according to Matthiessen’s rule:

(1) $$\tau_{total}^{-1}=\tau_{U}^{-1}+\tau_{N}^{-1}+\tau_{PD}^{-1}+\cdots$$

The fitting results are presented in Figure 6b. Compared with conventional Na-doped PbTe, the samples with Na, Mn, and Sn co-doping exhibit significantly lower lattice thermal conductivity. At high temperatures, the experimental data agree well with the model fitting, indicating that the dominant phonon scattering mechanisms are well captured. The analysis of the fitting results indicates that the incorporation of Na, Mn, and Sn effectively introduces point defect scattering, which scatters phonons and reduces the lattice thermal conductivity, whereas it also corroborates that the introduction of Sn facilitates the incorporation of a small amount of enriched Na into the lattice. The electronic and total thermal conductivities are presented in Figure S5a,b in the Supplementary Materials. Owing to the consistently low lattice thermal conductivity, the total thermal conductivity maintains a level of approximately 0.9 W m^−1^ K^−1^ at elevated temperatures.

**Figure 7:**
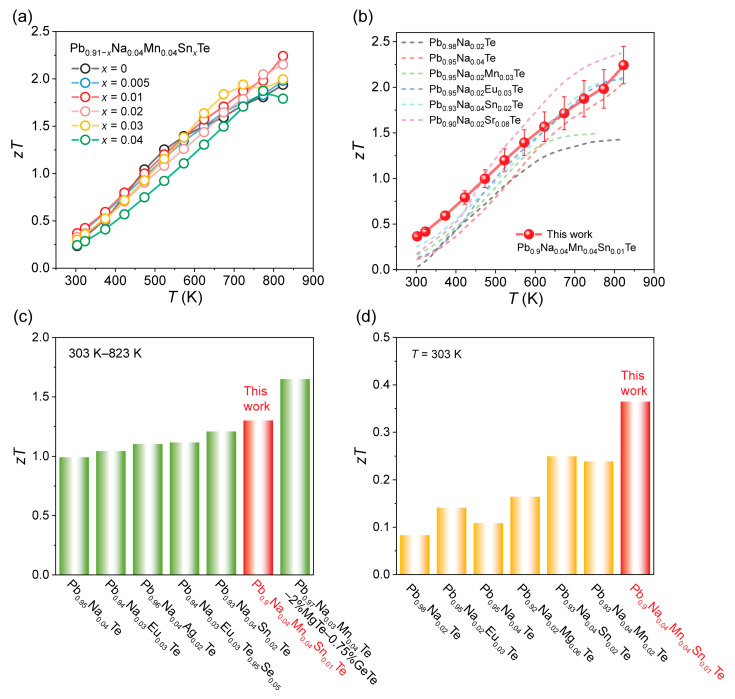
(**a**) Dimensionless figure of merit *zT* of Pb_0.91−*x*_Na_0.04_Mn_0.04_Sn*_x_*Te; the relative uncertainty of *zT* is ~10%. (**b**) The comparison of this work and partial reported values for *zT* [7,20,22,42,43]. (**c**) The comparison of average *zT* values from 303 K to 823 K. (**d**) The comparison of *zT* at 303 K [46,47,48,49].

Benefiting from the simultaneous enhancement in electrical and thermal transport properties, the thermoelectric figure of merit *zT* of the Pb_0.91-*x*_Na_0.04_Mn_0.04_Sn*_x_*Te series was evaluated with ~10% relative uncertainty (Table S3 in Supplementary Materials), and the results are presented in Figure 7a. Compared with the Sn-free matrix, all Sn-containing samples exhibit improved *zT* values. Figure 7b compares the performance of our samples with other Na-doped PbTe systems reported in the literature [42,43]. It is evident that our work achieves outstanding thermoelectric performance at both room and elevated temperatures. Notably, our material achieves an exceptional peak *zT* of approximately 2.2, together with an average *zT* exceeding 1.3 (Figure 7c). This outstanding performance is primarily attributed to the enhanced carrier effective mass resulting from Sn doping. To evaluate the thermoelectric performance of the material, the theoretical conversion efficiency was calculated based on the formula below [44,45]. The results reveal a remarkably high theoretical conversion efficiency of 15% (see Figure S6 in the Supplementary Materials).

(2) $$\eta_{max}=\frac{T_{h}-T_{c}}{T_{h}}\frac{\sqrt{1+{zT}_{avg}}-1}{\sqrt{1+{zT}_{avg}}+\frac{T_{c}}{T_{h}}}$$

A comprehensive comparison with other Na-doped PbTe systems is presented in Figure 7d. Relative to benchmark compositions such as Pb_0.95_Na_0.04_Te, Pb_0.93_Na_0.04_Sn_0.02_Te, and Pb_0.93_Na_0.04_Mn_0.02_Te [46,47,48,49], the room-temperature *zT* values achieved in this work rank among the highest. This enhancement is primarily attributed to the improved Seebeck coefficient arising from the band convergence enabled by the co-doping of Mn and Sn. Furthermore, the excellent stability of the material is confirmed by repeated measurements, as shown in Figure S7 in the Supplementary Materials.

## 4. Conclusions

In summary, we have developed a synergistic Mn and Sn co-doping strategy to simultaneously enhance the electrical and thermal transport properties of p-type PbTe. The incorporation of Sn induces additional Pb vacancies, increasing the hole carrier concentration while further promoting the dissolution of Na into the matrix, as evidenced by SEM and EDS analyses. More importantly, the co-doping of Mn and Sn significantly enhances the convergence between the *L* and *Σ* valence bands, leading to a substantial increase in the density-of-states effective mass and a marked enhancement in the Seebeck coefficient. Meanwhile, the multiscale defects introduced by the dopants − including point defects, dislocations, and nanoprecipitates—effectively scatter phonons across a wide frequency range, reducing the lattice thermal conductivity to near the theoretical minimum (~0.5 W m^−1^ K^−1^). Benefiting from these synergistic effects, the Pb_0.91-*x*_Na_0.04_Mn_0.04_Sn*_x_*Te system achieves an exceptional peak *zT* of ~2.2 at 823 K, a high room-temperature *zT* of ~0.4, and a favorable average *zT* of ~1.3 over the temperature range of 303–823 K. These results highlight that Mn and Sn co-doping provides an effective route for realizing both high peak and average thermoelectric performance in PbTe-based materials, offering a promising strategy for practical waste-heat recovery applications.

## Data Availability

The original contributions presented in this study are included in the article/Supplementary Material. Further inquiries can be directed to the corresponding author.

## Supplementary Materials

The following supporting information [50,51,52,53,54] can be downloaded at https://www.mdpi.com/article/10.3390/ma19101947/s1: Figure S1: The fitted Hall mobility with 3 m_e_ effective mass under the acoustic scattering assumption; the red dots represent the experimental data of the Pb_0.91−*x*_Na_0.04_Mn_0.04_Sn*_x_*Te sample. Figure S2: The resistivity of the Pb_0.91−*x*_Na_0.04_Mn_0.04_Sn*_x_*Te sample. Figure S3: The estimated specific heat capacity of the Pb_0.91−*x*_Na_0.04_Mn_0.04_Sn*_x_*Te sample. Figure S4: The measured thermal diffusivity coefficient of the Pb_0.91−*x*_Na_0.04_Mn_0.04_Sn*_x_*Te sample. Figure S5: (a) Electrical thermal conductivity and (b) total thermal conductivity of Pb_0.91−*x*_Na_0.04_Mn_0.04_Sn*_x_*Te. Figure S6: The calculated conversion efficiency of the Pb_0.91−*x*_Na_0.04_Mn_0.04_Sn*_x_*Te (*x* = 0.01, 0.02) sample. Figure S7: The retest of the Seebeck coefficient (*S*), resistivity (*ρ*), and thermal diffusivity (*D*) of the Pb_0.90_Na_0.04_Mn_0.04_Sn_0.01_Te sample. Table S1: The density of sintered Pb_0.91−*x*_Na_0.04_Mn_0.04_Sn*_x_*Te samples, which was measured by the Archimedes method. The value of the theoretical density is taken as 8.16 g cm^−3^ for PbTe. The calculation of density does not take the effect of porosity into account. Table S2: Parameters used to calculate *κ*_lat_ based on various phonon scattering processes via the modified Debye–Callaway model. Table S3: Peak *zT* of the sample and corresponding error.
