# Supplementary material for: Synergistic Sn-Induced Band Convergence in Mn-Doped p-Type PbTe Enables High Thermoelectric Performance
Source: Materials (Basel). 2026 May 9;19(10):1947. doi: 10.3390/ma19101947 (PMC13208535; doi:10.3390/ma19101947)
Supplement: Supplementary file 1 [file materials-19-01947-s001.zip › materials-4264352-supplementary.pdf]

Supplementary Information

Synergistic Sn-Induced Band Convergence in Mn-Doped p-Type PbTe Enables High Thermoelectric Performance

Zhilong Zhao [1,†], Xiang An [1,†], Fan Feng [1], Jiaxing Luo [1], Zijian Lin [1], Chuke Zhao [1] and Ran Ang [1,2,3,*]

[1] Key Laboratory of Radiation Physics and Technology, Ministry of Education, Institute of Nuclear Science and Technology, Sichuan University, Chengdu 610064, China; zhao@stu.scu.edu.cn (Z.Z.); axwinner@163.com (X.A.); ff001025@163.com (F.F.); gnixaijl@163.com (J.L.); 13970966083@163.com (Z.L.); zchuke2000@163.com (C.Z.)

[2] College of Physics, Sichuan University, Chengdu 610064, China

[3] Institute of New Energy and Low-Carbon Technology, Sichuan University, Chengdu 610065, China

† These authors contribute to this work equally.

* Correspondence: rang@scu.edu.cn

Modelling

Single parabolic band (SPB)

The corresponding thermoelectric properties are derived using the below equations.

The Seebeck coefficient S:

$$S = \frac{k_B}{e}\left(\frac{2F_1(\eta)}{F_0(\eta)} - \eta\right) \tag{S1}$$

where η is the reduced chemical potential (E_F/k_BT), k_B is the Boltzmann constant, and e is the electron charge.

The carrier concentration n_H:

$$n_H = 4\pi\left[\frac{2m^* k_B T}{h^2}\right]^{\frac{3}{2}} F_{1/2}(\eta) \tag{S2}$$

where m^* is the density of state effective mass, h is Plank's constant, and T is the absolute temperature.

The mobility μ_H:

$$\mu_H = \frac{e}{m^*}\frac{\pi\hbar^4 v_L^2 \rho}{\sqrt{2}E_{def}^2 (m^* k_B T)^{\frac{3}{2}}}\frac{F_{1/2}(\eta)}{2F_0(\eta)} \tag{S3}$$

where u_L is the longitudinal sound velocity, ρ is the density of the sample, and E_{def} is a combination of deformation potentials for multivalley systems [3].

The Hall factor r_H:

$$r_H = \frac{3}{2}F_{1/2}(\eta)\frac{F_{-1/2}(\eta)}{2F_0^2(\eta)} \quad \text{(S4)}$$

The Hall factor reflects the energy scattering mechanism and the anisotropy of the energy band. For the SPB model, anisotropy does not need to be considered. The Lorenz number L:

$$L = \frac{\kappa_B^2}{e^2}\frac{3F_0(\eta)F_2(\eta) - 4F_1^2(\eta)}{F_0^2(\eta)} \quad \text{(S5)}$$

In the equations above, the integral F_j is defined by

$$F_j(\eta) = \int_0^{\infty}\frac{\xi^j d\xi}{1+e^{(\xi-\eta)}} \quad \text{(S6)}$$

In this work, the effective mass (m^*) will be determined with experimental n_H and the Seebeck coefficient using Equations S1-S2.

Two-band model

Assuming that the light band is non-parabolic and the heavy band is parabolic, the SKB (single Kane band) model and SPB mode are applied for light bands and heavy bands respectively[4-6]. It should be noted that the rigid band approximation is adopted which assumes that the changing carrier concentration adjusts only the chemical potential position and not the shape or position of the bands. As for the single Kane band (SKB):

The Seebeck coefficient S:

$$S = \frac{\mathrm{k_B}}{e}\left[\frac{\mathrm{F}_{1,1}^{-2}}{\mathrm{F}_{1,0}^{-2}} - \eta\right] \quad \text{(S7)}$$

The carrier concentration n_H:

$$n_{\mathrm{H}} = \frac{1}{A}\left[\frac{2m^*k_{\mathrm{B}}T}{\hbar^2}\right]^{\frac{3}{2}}\mathrm{F}_{2/3,0}^{0} \quad \text{(S8)}$$

The mobility μ_H:

$$\mu = \frac{2\pi\hbar^4 eC_l}{\mathrm{m}_I^*(2m^*k_BT)^{\frac{3}{2}}\Xi^2}\frac{3\mathrm{F}_{1,0}^{-2}}{\mathrm{F}_{3/2,0}^{0}} \quad \text{(S9)}$$

Due to the anisotropy of both conduction and valence bands, the inertial effective mass m_I^* and the density of states effective mass m^* are governed by

the effective band mass of a single pocket along two directions $m_{\parallel}^*$ and $m_{\perp}^*$:

$$m^* = N_V^{\frac{2}{3}} m_b^* = N_V^{\frac{2}{3}} \left(m_{\perp}^{*2} m_{\parallel}^{*2}\right)^{\frac{1}{3}};\ m_I^* = 3\left(\frac{2}{m_{\perp}^*} + \frac{1}{m_{\parallel}^*}\right)^{-1} \tag{S10}$$

where N_V is the band degeneracy (N_{V1}=4 for the light-mass valence band; N_{V2}=12 for the heavy-mass valence band of PbTe).

The Hall factor R_H:

$$R_H = \frac{3K(K+2)}{(2K+1)^2} \frac{F^0_{\frac{1}{2},-4} F^0_{\frac{3}{2},0}}{F^0_{1,-2}} \tag{S11}$$

where $K = m_{\parallel}^*/m_{\perp}^*$ (K=1, assumed T-independent), which reflects the anisotropy of the energy band.

In the equations above, the integral $F_{m,n}^l$ is defined by

$$\mathrm{F}^l_{m,n}(\eta,\alpha) = \int_0^\infty \left(-\frac{\partial f}{\partial \varepsilon}\varepsilon^n\right)(\varepsilon + \varepsilon^2\alpha)^m (1 + 2\varepsilon\alpha)^l \mathrm{d}\varepsilon \tag{S12}$$

where $\alpha = \frac{\mathrm{k}_B T}{E_g}$ ($\mathrm{E_g}$ is the band gap) is the reciprocal reduced band gap and ε is the reduced carrier energy. The Lorenz number (L) was estimated using the SKB model.

The total electrical conductivity:

$$\sigma = \sigma_L + \sigma_\Sigma \tag{S13}$$

The total Seebeck coefficient:

$$S = \frac{S_L\sigma_L + S_\Sigma\sigma_\Sigma}{\sigma_L + \sigma_\Sigma} \tag{S14}$$

The total Lorenz number:

$$L = \frac{L_L\sigma_\mathrm{L} + L_\Sigma\sigma_\Sigma}{\sigma_L + \sigma_\Sigma} \tag{S15}$$

The total carrier concentration n_H:

$$n_H = \frac{[bn_{L\mathrm{H}} + n_{\Sigma H}]^2}{A_{LH} b^2 n_{L\mathrm{H}} + A_{\Sigma H n_{\Sigma H}}} \tag{S16}$$

where b = 4, which is suitable for the PbTe system.

The total Hall coefficient R_H:

$$R_H = \frac{\sigma_L^2 R_{LH} + \sigma_\Sigma^2 R_{\Sigma H}}{(\sigma_L + \sigma_\Sigma)^2} \tag{S17}$$

The total Hall mobility μ_H:

$$\mu_H = R_{\mathrm{H}}\sigma \tag{S18}$$

Debye–Callaway model simulation of lattice thermal conductivity k_{lat}

According to the Debye–Callaway model[7,8], κ_L can be calculated by

$$\kappa_L = \frac{k_B}{2\pi^2 v}\left(\frac{k_B}{\hbar}\right)^3 \int_0^{\Theta/T} \tau(x)\frac{x^4 e^x}{(e^x - 1)^2}dx \tag{S19}$$

where v=$3^{1/3}(v_L^{-3}+2\ v_T^{-3})^{-1/3}$ (with v_T and v_L respectively denoting the transverse and longitudinal sound velocities) is the average speed of a phonon, $x = \hbar\omega/k_B T$ (with ω denoting the phonon frequency) is the reduced phonon frequency, Θ is the Debye temperature, $\hbar$ is the reduced Planck constant, k_B is the Boltzmann constant, and τ_{tot} is the total relaxation time. The phonon scattering pathways include phonon–phonon Umklapp (U), normal processes (N), point defects (PDs), and precipitates (Ps). The relevant phonon relaxation times are given by:

Umklapp phonon scattering and normal process:

$$\tau_U^{-1} = \frac{\hbar\omega^2\gamma^2 T}{M v^2 \Theta}e^{-\Theta/T} \tag{S20}$$

The relaxation time influenced by the normal process can be simplified as:

$$\tau_N^{-1} = \beta\tau_U^{-1} \tag{S21}$$

Point defect phonon scattering:

$$\tau_{PD}^{-1} = \frac{V_0}{4\pi^2 v}\Gamma\omega^4 \tag{S22}$$

$$\Gamma = \frac{\sum_{k=1}^{n} c_k\left(\frac{\overline{M_k}}{\overline{\overline{M}}}\right)^2 f_k^1 f_k^2 \varepsilon\left(\frac{M_k^1 - M_k^2}{\overline{M_k}}\right)^2}{\left(\sum_{k=1}^{n} c_k\right)} + \frac{\sum_{k=1}^{n} c_k\left(\frac{\overline{M_k}}{\overline{\overline{M}}}\right)^2 f_k^1 f_k^2 \varepsilon\left(\frac{r_k^1 - r_k^2}{\overline{r_k}}\right)^2}{\left(\sum_{k=1}^{n} c_k\right)} \tag{S23}$$

Precipitate scattering:

$$\tau_P^{-1} = v({\sigma_s}^{-1} + {\sigma_L}^{-1})^{-1}V_p \tag{S24}$$

In the above equations, M is the average atomic mass, γ is the Grüneisen parameter, Θ is the Debye temperature, β is the ratio between the normal process and Umklapp phonon scattering, V_0 is the average atomic volume, v is the average sound velocity, v_L is the longitudinal velocity, v_T is the transverse velocity, and Γ is the point defect scattering parameter. Herein, we used the parameters of PbTe to calculate κ_{lat}. v, v_L, v_T, Θ and γ were determined from the

reference, M; V_0 was determined from the calculated properties; and ρ was measured by the Archimedes method. All these related parameters used for the calculation are listed in Table S2.

Statistical analysis

Electrical and thermal transport properties were measured using a commercial CTA Pro system and a Netzsch LFA 467 Pro instrument, respectively. Since the measurement results are largely insensitive to sample dimensional variations, the primary source of uncertainty arises from the instrumental standard deviations. Specifically, the systematic uncertainties in the Seebeck coefficient (S) and electrical conductivity (σ) are estimated to be approximately 3% and 5%, respectively. The relative errors of the derived parameters were evaluated using standard error propagation formulas (S24).

$$u_{rel} = \frac{u_c(q)}{|q|} = \sqrt{\sum_{i=1}^{n} \left[\frac{\partial lnf}{\partial x_i} u_c(x_i)\right]^2} \tag{S25}$$

The relative uncertainties in sample density, specific heat capacity, and thermal diffusivity are approximately 1%, 5%, and 5%, respectively. The relative error of the total thermal conductivity was accordingly calculated using S25, and the resulting relative error of the thermoelectric figure of merit zT was obtained via S26. The revised content has been included in the updated Supplementary Information.

$$\frac{d\kappa_{tot}}{\kappa_{tot}} = \sqrt{\left(\frac{d\rho}{\rho}\right)^2 + \left(\frac{dC_P}{C_P}\right)^2 + \left(\frac{dD}{D}\right)^2} \tag{S26}$$

$$\frac{dzT}{zT} = \sqrt{\left(2 \times \frac{dS}{S}\right)^2 + \left(\frac{d\sigma}{\sigma}\right)^2 + \left(\frac{d\kappa_{tot}}{\kappa_{tot}}\right)^2} \tag{S27}$$

Supplementary figure

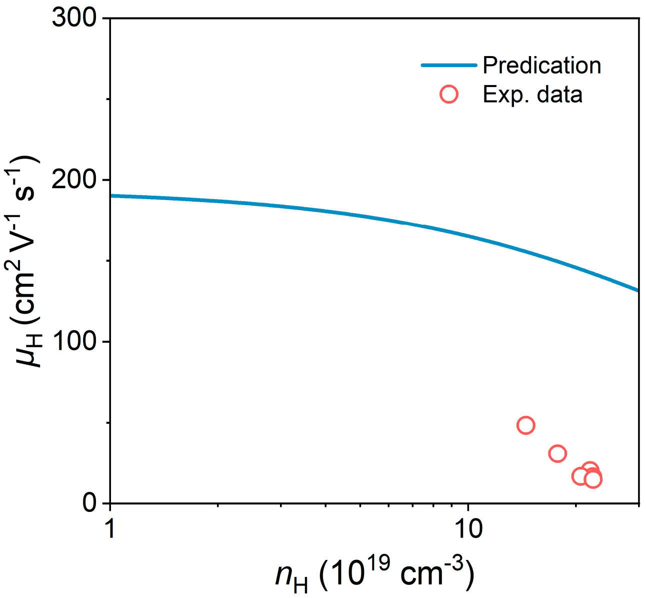

Figure S1. The fitted Hall mobility with 3 m_e effective mass under the acoustic scattering assumption; the red dots represent the experimental data of the $Pb_{0.91-x}Na_{0.04}Mn_{0.04}Sn_xTe$ sample.

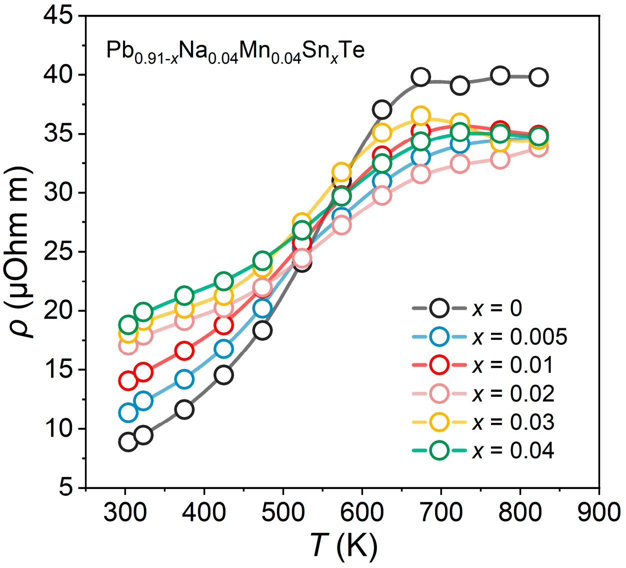

Figure S2. The resistivity of the $Pb_{0.91-x}Na_{0.04}Mn_{0.04}Sn_xTe$ sample.

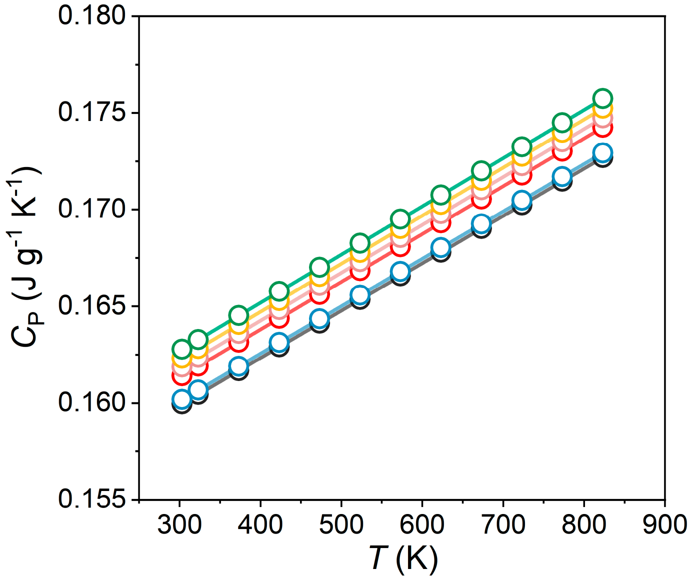

Figure S3. The estimated specific heat capacity of the $Pb_{0.91-x}Na_{0.04}Mn_{0.04}Sn_xTe$ sample.

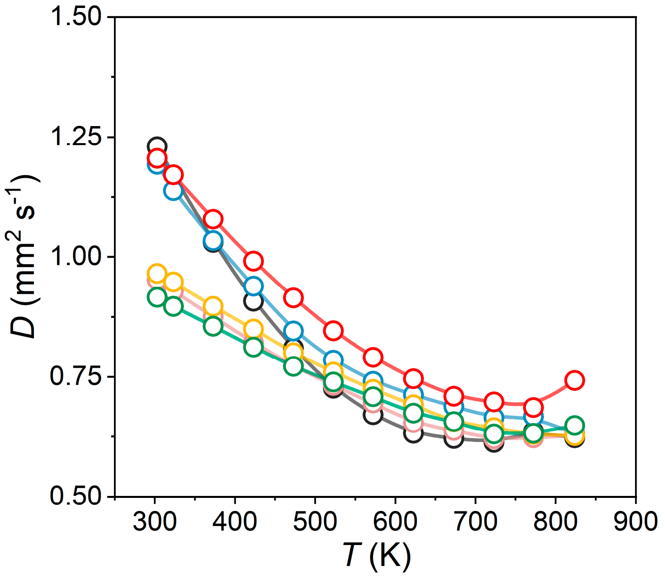

Figure S4. The measured thermal diffusivity coefficient of the $Pb_{0.91-x}Na_{0.04}Mn_{0.04}Sn_xTe$ sample.

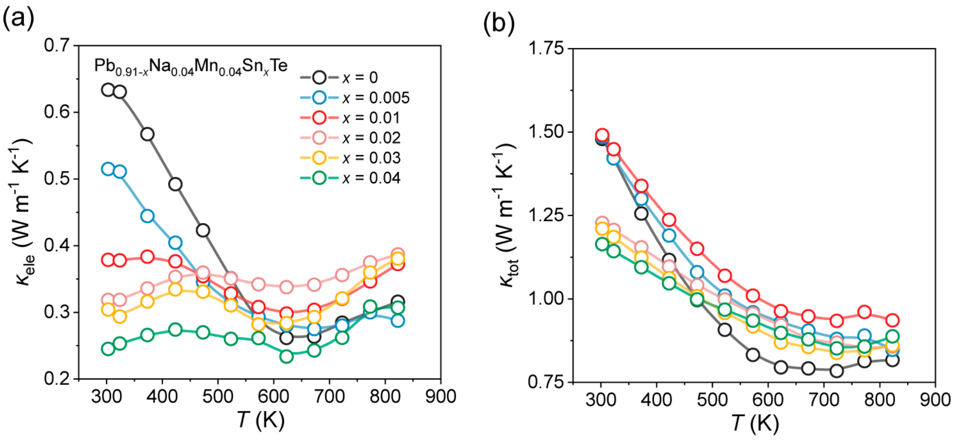

Figure S5. (a) Electrical thermal conductivity and (b) total thermal conductivity of $Pb_{0.91-x}Na_{0.04}Mn_{0.04}Sn_xTe$.

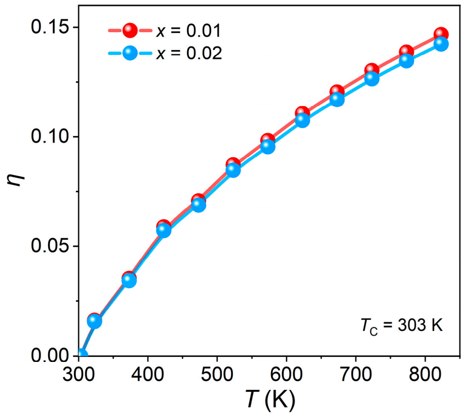

Figure S6. The calculated conversion efficiency of the $Pb_{0.91-x}Na_{0.04}Mn_{0.04}Sn_xTe$ (x = 0.01, 0.02) sample.

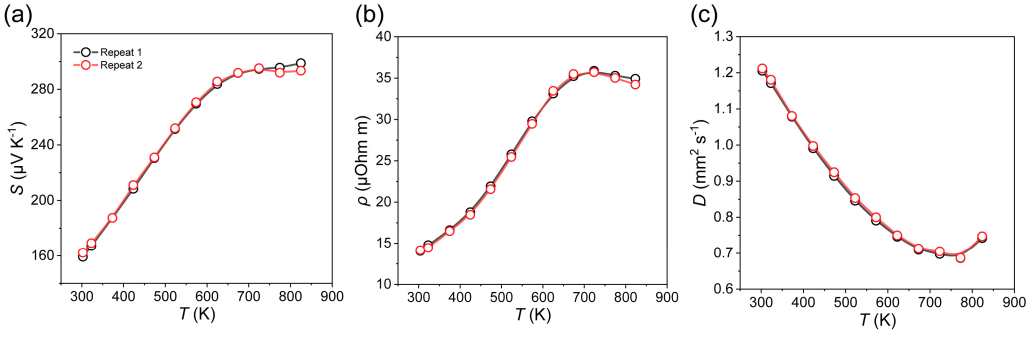

Figure S7. The retest of the Seebeck coefficient (S), resistivity (ρ), and thermal diffusivity (D) of the $Pb_{0.90}Na_{0.04}Mn_{0.04}Sn_{0.01}Te$ sample.

Supplementary Table

Table S1. The density of sintered $Pb_{0.91-x}Na_{0.04}Mn_{0.04}Sn_xTe$ samples, which was measured by the Archimedes method. The value of the theoretical density is taken as 8.16 for PbTe. The calculation of density does not take the effect of porosity into account.

Sample	Measured density g cm^{-3}	Relative density %
$x = 0$	7.74	94.9
$x = 0.005$	7.77	95.2
$x = 0.01$	7.76	95.1
$x = 0.02$	7.68	94.1
$x = 0.03$	7.62	93.4
$x = 0.04$	7.60	93.1

Table S2. Parameters used to calculate κ_{lat} based on various phonon scattering processes via the modified Debye–Callaway model.

Parameters	Value
Lattice constant *a*, *b* and *c* (Å)	a = b = c = 6.41
Average atomic mass *M* (kg)	2.74092×10^{-25}
Average atomic mass volume V_0 (m^3)	3.35733×10^{-29}
Boltzmann constant k_B (J/K)	1.38×10^{-23}
Grüneisen parameter γ	1.86
Point defect scattering parameter Γ	2.0337×10^{-1}
Average sound velocity v (m/s)	1770
Longitudinal velocity v_L (m/s)	3590
Transverse velocity v_T (m/s)	1260
Debye temperature Θ (K)	136
Ratio of N to U processes β	2.5

Table S3. Peak *zT* of the sample and corresponding error.

Sample	Peak *zT*	Error
$x = 0$	1.93	±0.193
$x = 0.005$	1.97	±0.197
$x = 0.01$	2.24	±0.224
$x = 0.02$	2.15	±0.215
$x = 0.03$	1.99	±0.199
$x = 0.04$	1.79	±0.179

References

1. Gibbs, Z.M.; Ricci, F.; Li, G.; Zhu, H.; Persson, K.; Ceder, G.; Hautier, G.; Jain, A.; Snyder, G.J. Effective Mass and Fermi Surface Complexity Factor from Ab Initio Band Structure Calculations. *npj Comput. Mater*. **2017**, 3, 8.
2. Bessas, D.; Sergueev, I.; Wille, H.-C.; Perßon, J.; Ebling, D.; Hermann, R.P. Lattice Dynamics in $Bi_2Te_3$and Sb_2Te_3: Te and Sb Density of Phonon States. *Phys. Rev. B* **2012**, 86, 224301.
3. Jang, H.; Park, J.H.; Lee, H.S.; Ryu, B.; Park, S.-D.; Ju, H.-A.; Yang, S.-H.; Kim, Y.-M.; Nam, W.H.; Wang, H.; et al. Regulating Te Vacancies through Dopant Balancing via Excess Ag Enables Rebounding Power Factor and High Thermoelectric Performance in P-Type PbTe. *Adv. Sci.* **2021**, 20, 2100895
4. Herring, C.; Vogt, E. Transport and Deformation-Potential Theory for Many-Valley Semiconductors with Anisotropic Scattering. *Phys. Rev.* **1956**, 101, 944–961.
5. Callaway, J.; von Baeyer, H.C. Effect of Point Imperfections on Lattice Thermal Conductivity. *Phys. Rev.* **1960**, 120, 1149–1154.
